# Association of serum adalimumab levels and immunogenicity with clinical response in non-infectious uveitis: a real-world cohort study

**DOI:** 10.3389/fimmu.2026.1855538

**Published:** 2026-07-20

**Authors:** Pau Otal, Julia Seco-Orriols, Lucía Miguel-Escuder, Aina Moll-Udina, Maite Sainz-de-la-Maza, Ignasi Rodríguez-Pintó, Mireia Hereu, Juan P. Codes, Alfredo Adán, Víctor Llorenç

**Affiliations:** 1 Ocular Inflammation and Infection Section, Department of Ophthalmology, Hospital Clínic de Barcelona, Barcelona, Spain; 2 Institut d’Investigacions Biomèdiques August Pi i Sunyer (IDIBAPS), Barcelona, Spain; 3 Department of Autoimmune Diseases, Reference Centre for Systemic Autoimmune Diseases (UEC, CSUR) of the Catalan and Spanish Health Systems/Member of European Reference Network (ERN)-ReCONNET, Hospital Clínic, Barcelona, Catalonia, Spain

**Keywords:** adalimumab, anti-adalimumab antibodies, immunogenicity, non-infectious uveitis, pharmacokinetics, therapeutic drug monitoring, uveitis

## Abstract

**Purpose:**

To evaluate serum adalimumab (ADA) levels in patients with non-infectious uveitis (NIU), identify factors associated with drug exposure—including anti-adalimumab antibodies (AAA)—and assess their relationship with control of intraocular inflammation.

**Methods:**

This retrospective, observational, single-center study included patients with NIU treated with ADA who underwent at least one proactive assessment of serum ADA levels and AAA between October 2019 and January 2024 at Hospital Clínic de Barcelona (Spain).

**Results:**

A total of 135 measurements were obtained from 65 patients. Mean serum ADA levels were higher in patients with complete response (8.28 µg/mL) compared with partial responders (5.57 µg/mL; p = 0.016) and non-responders (6.21 µg/mL; p = 0.012). AAA were detected in 18/135 measurements (13.3%). Patients with detectable AAA had markedly lower serum ADA levels (1.44 µg/mL; p < 0.0001). Lower ADA levels were also associated with male sex (p = 0.011), obesity (body mass index >30 kg/m²; p = 0.006), panuveitis (p < 0.0001), presence of vitritis (p = 0.0002), and longer disease duration (p < 0.0001). No significant differences were observed between ADA monotherapy and combination therapy with conventional immunomodulators (p = 0.91).

**Conclusions:**

In NIU, patients achieving complete inflammatory control showed higher serum ADA levels than those with partial response or persistent disease activity, suggesting an association between drug exposure and inflammatory control. ADA exposure is influenced by patient- and disease-related factors, including body mass index, inflammatory burden, anatomical classification, immunogenicity, and dosing strategy. These findings support a potential role for proactive therapeutic drug monitoring to guide individualized treatment strategies.

## Introduction

Uveitis comprises a heterogeneous group of intraocular inflammatory disorders, with an estimated prevalence ranging from 38 to 204 cases per 100, 000 inhabitants in Western countries. It accounts for up to 30% of visual impairment and between 2.8% and 10% of preventable blindness (1, 2). Non-infectious uveitis (NIU) is frequently associated with systemic immune-mediated diseases and represents a major cause of morbidity among individuals of working age.

The pathogenesis of NIU involves dysregulated immune responses affecting both innate and adaptive pathways. Tumor necrosis factor alpha (TNF-α) plays a central role in this process by promoting leukocyte activation, endothelial adhesion, and disruption of the blood–ocular barrier, thereby sustaining intraocular inflammation (3). Targeting TNF-α has therefore become an established therapeutic strategy. Adalimumab (ADA), a fully human monoclonal IgG1 antibody, neutralizes both soluble and membrane-bound TNF-α and inhibits its interaction with TNF receptors p55 and p75 (4, 5). It is currently the only anti-TNF agent approved by both the Food and Drug Administration and the European Medicines Agency for the treatment of non-anterior NIU.

The efficacy and safety of ADA have been demonstrated in randomized controlled trials, including the VISUAL I and VISUAL II studies, which showed a reduced risk of treatment failure in patients with active disease and in those with corticosteroid-dependent inactive NIU (6, 7). These findings are supported by real-world data reporting sustained control of ocular inflammation in approximately 65–87% of patients during long-term follow-up (8, 9). However, a substantial proportion of patients either fail to respond initially or lose response over time, highlighting the need for improved treatment optimization strategies.

Pharmacokinetic variability and immunogenicity are key factors influencing the response to monoclonal antibodies. ADA has a half-life of approximately two weeks, although drug exposure varies considerably between individuals (10, 11). Subtherapeutic drug levels may result in incomplete TNF-α blockade and persistent inflammatory activity. In other immune-mediated diseases, including rheumatoid arthritis, psoriasis, and inflammatory bowel disease, multiple studies have demonstrated a strong association between serum ADA levels and clinical outcomes, supporting the use of therapeutic drug monitoring (TDM) to guide treatment decisions (12–14). More recently, proactive TDM strategies have been associated with improved long-term disease control and cost-effectiveness by enabling early dose optimization and reducing the risk of treatment failure (15, 16).

In NIU, the role of TDM is less clearly defined. Emerging evidence suggests that adequate serum ADA levels are associated with better control of ocular inflammation, although a well-established therapeutic range is still lacking (17–19). Recent studies have further highlighted the potential clinical value of TDM in uveitis, suggesting that dose adjustments based on serum ADA levels and anti-adalimumab antibodies (AAA) status may improve outcomes and reduce relapse rates (20–23). In addition, prospective and translational data indicate that early drug exposure may influence long-term immunogenicity and treatment durability, supporting a more individualized treatment approach (21–24).

Immunogenicity represents another important determinant of ADA efficacy. The development of AAA can increase drug clearance and reduce bioavailability, ultimately compromising treatment effectiveness. Despite being a fully human antibody, ADA is associated with AAA formation in a considerable proportion of patients, with reported rates ranging from 5% to 64% across immune-mediated diseases (10, 25). The presence of AAA has consistently been linked to lower serum ADA levels and poorer clinical outcomes (25). Mechanistically, AAA may neutralize ADA activity or promote the formation of immune complexes that accelerate drug clearance.

Recent data in uveitis further support the clinical relevance of immunogenicity. Studies have shown that AAA positivity is associated with reduced treatment response, increased relapse rates, and a higher likelihood of treatment discontinuation (20–24). Several factors have been associated with AAA development, including low drug exposure, intermittent dosing, and the absence of concomitant immunosuppressive therapy (21–23). The use of concomitant immunomodulators, such as methotrexate, has been shown to reduce immunogenicity in other immune-mediated diseases, although its role in NIU remains to be fully established (26).

Despite these advances, current evidence in NIU is limited by small sample sizes, heterogeneous study designs, and the lack of standardized assays for ADA and AAA measurement. Most available data derive from retrospective cohorts or small prospective studies, and only a limited number of multicenter analyses have been reported (17–24). In addition, variability in laboratory methods complicates the definition of clinically relevant thresholds for ADA levels and AAA detection.

Taken together, these limitations underscore the need for a more individualized approach to biologic therapy in NIU. Integrating pharmacokinetic and immunogenicity data into clinical decision-making may help optimize treatment efficacy while minimizing unnecessary drug exposure. In this context, TDM represents a promising tool to support precision medicine in uveitis.

In this study, we present a single-center retrospective analysis of a cohort of adult patients with NIU who underwent at least one measurement of serum ADA and AAA levels. Therefore, our aim was to evaluate serum ADA levels in patients with NIU, identify factors associated with drug exposure—including AAA—and assess their relationship with control of intraocular inflammation.

## Methods

### Study design and patients

This retrospective, observational, single-center study included adult patients with non-infectious uveitis (NIU) treated with adalimumab (ADA) who underwent at least one assessment of serum ADA levels and anti-adalimumab antibodies (AAA). Therapeutic drug monitoring (TDM) was performed as part of a proactive clinical strategy during routine follow-up visits, at predefined intervals of 3 to 6 months, irrespective of uveitis activity at the time of sampling. However, because of the retrospective nature of the study, the final indication and specific timing of each test were subject to the treating physician’s discretion.

Patients were recruited from the Ocular Inflammation Section, Department of Ophthalmology, Hospital Clínic de Barcelona, between October 2019 and January 2024. Patients receiving ADA for indications other than NIU were excluded.

The study protocol was approved by the local Ethics Committee (HCB/2021/0437), which waived the requirement for informed consent due to the retrospective design. The study adhered to the tenets of the Declaration of Helsinki (2013 revision).

This study was reported in accordance with the Strengthening the Reporting of Observational Studies in Epidemiology (STROBE) guidelines for cohort studies.

Regarding the selection criteria, covariates were chosen based on two main principles:

1. Clinical and pathophysiological relevance: Known factors in ophthalmological practice that reflect disease severity (e.g., vitreous haze, uveitis type, disease duration);
2. Pharmacokinetic plausibility: Factors widely documented in literature to influence anti-TNF drug clearance, immunogenicity, or serum concentrations (e.g., Body Mass Index, concomitant immunosuppression, and prior treatment exposure).

### Adalimumab treatment

ADA (Humira^®^, AbbVie Inc.) was initiated in patients with active NIU refractory to conventional immunomodulatory therapy (IMT) or in cases of intolerance to IMT. When clinically appropriate, concomitant IMT (e.g., methotrexate) was maintained or added to reduce immunogenicity.

ADA was administered subcutaneously according to the approved dosing regimen: an induction dose of 80 mg at baseline, followed by 40 mg at week 1, and subsequently 40 mg every other week. Dose adjustments were made based on clinical response. In patients with sustained disease control after 6–12 months, treatment optimization consisted of extending the dosing interval (≥3 weeks). In cases of inadequate response, dose intensification to 40 mg weekly was implemented.

### Clinical evaluation

All patients underwent standardized ophthalmological assessment, including best-corrected visual acuity, slit-lamp bio microscopy, and dilated fundus examination. Inflammatory activity was graded using the Standardization of Uveitis Nomenclature (SUN) criteria for anterior chamber cells and the National Eye Institute (NEI) scale for vitreous haze (25, 26).

Macular edema was defined as a central retinal thickness (CRT) >315 µm with the presence of intraretinal and/or subretinal fluid on spectral-domain optical coherence tomography (SD-OCT) (27). OCT imaging was performed using Cirrus HD-OCT^®^ (Carl Zeiss Meditec, USA).

Ultra-widefield fluorescein angiography (FA) (Optos^®^, UK) was performed in cases of suspected retinal vasculitis, while indocyanine green angiography (ICGA) was used when choroidal involvement was suspected.

### Definition of treatment response

Treatment response was classified into three categories based on multimodal assessment, adapted from SUN definitions (25) and previous studies: Complete response: absence of inflammatory cells in the anterior chamber and vitreous (grade 0), absence of macular edema, and no signs of active inflammation on clinical examination, OCT, FA, or ICGA. Partial response: improvement without complete resolution, defined as any of the following: ≥2-step decrease in anterior chamber or vitreous inflammation with residual activity ≥0.5+, ≥20% reduction in CRT with CRT ≥316 µm, or reduction in angiographic activity without complete quiescence. No response: persistence of active inflammation, including intraocular cells, active chorioretinal lesions, angiographic leakage, or macular edema not meeting partial response criteria. Primary non-response was defined as the absence of a partial or complete clinical response within the first 3 months after initiation of adalimumab treatment. Secondary loss of response was defined as the recurrence or worsening of intraocular inflammation following an initial clinical response (partial or complete) to adalimumab.

### Data collection

The following variables were systematically collected: demographic characteristics, duration of follow-up, indication for ADA initiation, reason for TDM assessment, ADA dose and administration interval, anatomical classification of uveitis (SUN), laterality, associated systemic disease, previous and concomitant treatments, inflammatory status at the time of ADA/AAA measurement, and imaging findings (macular edema, vasculitis, choroiditis). Uveitis anatomical classification was evaluated as a proxy for potential variations in inflammatory burden based on disease extension.

Pharmacokinetic and immunogenicity data included serum ADA levels (µg/mL), presence and concentration of AAA (AU/mL), and time from ADA initiation to sampling.

All patients underwent a standardized diagnostic work-up to exclude infectious or masquerade uveitis. Prior to ADA initiation, patients were evaluated by internal medicine specialists and screened for chronic infections, including hepatitis B and C, human immunodeficiency virus, and tuberculosis (QuantiFERON^®^-TB Gold assay).

### Measurement of serum ADA levels and anti-adalimumab antibodies

Blood samples were collected immediately before ADA administration to determine trough levels. Serum ADA levels and AAA were measured using commercially available ELISA-based assays (Promonitor^®^-ADL and Promonitor^®^-anti-ADL; Progenika Biopharma, Spain).

These assays are based on sandwich enzyme-linked immunosorbent techniques and were performed according to the manufacturer’s instructions. ADA concentrations were expressed in µg/mL. AAA positivity was defined using a cut-off value of ≥10 arbitrary units (AU/mL), according to the manufacturer.

### Endpoints and statistical analysis

The primary endpoint was to identify factors associated with serum ADA levels. The secondary endpoint was to evaluate the relationship between clinical outcomes and serum ADA levels in patients with NIU.

Study covariates were classified according to their mathematical nature and clinical relevance, in accordance with STROBE recommendations. Serum ADA levels were analyzed as a continuous quantitative variable. Demographic, anthropometric, clinical, ophthalmological, and therapeutic covariates were analyzed as continuous, dichotomous, nominal, or ordinal variables, as appropriate. Continuous variables were retained as continuous in the multivariable linear regression mixed-effects models to avoid loss of statistical power, whereas categorical variables were entered using dummy variables where appropriate.

A formal sample size calculation was not performed due to the retrospective and exploratory nature of the study. Descriptive statistics were used to summarize patient characteristics. Categorical variables were expressed as absolute frequencies and percentages, while continuous variables were reported as mean ± standard deviation or median and interquartile range, depending on data distribution.

Comparisons between groups were performed using Student’s t-test or the Mann–Whitney U test for continuous variables, and the chi-square or Fisher’s exact test for categorical variables. For multiple group comparisons, Kruskal–Wallis tests were applied as appropriate. Disease evolution time was defined as the time elapsed from uveitis diagnosis to therapeutic drug monitoring assessment. For multiple pairwise comparisons, Bonferroni correction was applied.

Because some patients contributed more than one therapeutic drug monitoring assessment, the multivariable analysis was performed using a linear mixed-effects models (LMM), with patient identity included as a random effect to account for interpatient correlation. Serum adalimumab concentration was analyzed as a continuous dependent variable. Candidate covariates were initially evaluated in univariable analyses, and variables associated with serum adalimumab levels (p ≤ 0.1) were considered for inclusion in the multivariable model. Results are reported as regression coefficients, or β coefficients, with 95% confidence intervals and p-values.

Analyses were performed at the measurement level unless otherwise specified. No imputation of missing data was performed, as no relevant missing data were identified for the variables included in the analysis.

All analyses were conducted using R statistical software (version 3.5.3; R Foundation for Statistical Computing) and GraphPad Prism (version 9.2.0; GraphPad Software, San Diego, CA, USA). A two-sided p-value <0.05 was considered statistically significant.

Given the retrospective design, potential sources of bias include selection bias and variability in follow-up intervals.

## Results

### Patient characteristics and uveitis profile

A total of 65 patients with non-infectious uveitis (NIU) were included, contributing 135 measurements of serum adalimumab (ADA) levels and anti-adalimumab antibodies (AAA) between October 2019 and January 2024.

The mean age at the time of sampling was 44 years (range, 18–84), and 56.9% of patients were female. Most patients were Caucasian (80%), followed by those of Latin American origin (15.4%). The mean duration of follow-up from uveitis diagnosis was 81.7 months (range, 2–209), while the mean time from ADA initiation to sampling was 33.4 months (range, 1–168).

Uveitis was bilateral in 75.4% of patients, and 46.1% had an associated systemic immune-mediated disease. According to anatomical classification, cases were distributed as anterior (n=8), intermediate (n=5), posterior (n=25), and panuveitis (n=27), with four cases remaining etiologically unclassified.

At the time of drug monitoring, ADA was used as monotherapy in 54.1% of measurements. Concomitant treatments included systemic corticosteroids (n=31), immunomodulatory therapy (IMT; most commonly methotrexate, n=21), and combined IMT plus corticosteroids (n=10).

At the time of sampling, 66.7% of cases were classified as complete response, 19.2% as partial response, and 14.1% as no response. ADA dosing had been modified in a subset of cases, including dose spacing (18.5%) and dose intensification (13.3%) (**Table 1**).

**Table 1:** Demographic and clinical characteristics of patients with non-infectious uveitis undergoing adalimumab therapeutic drug monitoring.

Variable	Value
Age, median (range), years	44 (18–84)
Female sex, n (%)	37 (56.9)
Geographic origin, n (%)	
Caucasian	52 (80.0)
Latin American	10 (15.4)
Other	3 (4.6)
Anatomical classification, n (%)	
Anterior uveitis	8 (12.3)
Intermediate uveitis	5 (7.7)
Posterior uveitis	25 (38.5)
Panuveitis	27 (41.5)
Bilateral involvement, n (%)	49 (75.4)
Etiology, n (%)	
Systemic autoimmune disease-associated*	30 (46.1)
Birdshot chorioretinopathy	12 (18.4)
Behçet’s disease	10 (15.4)
Punctate inner choroiditis	6 (9.2)
VKH / sympathetic ophthalmia	12 (18.4)
Juvenile idiopathic arthritis	6 (9.2)
Serpiginous choroiditis	4 (6.2)
Unclassified	4 (6.2)
Previous treatment before adalimumab, n (%)	
Immunomodulatory therapy (IMT)	26 (40.0)
Prior anti-TNF therapy (infliximab, certolizumab, etanercept, golimumab):	6 (9.2)
IMT + prior non-anti-TNF biologic therapy (tocilizumab, rituximab, secukinumab)	14 (21.5)
Corticosteroids only	19 (29.3)
Concomitant therapy at time of TDM (n = 135 measurements), n (%)	
Methotrexate (MTX)	19 (14.1)
Mycophenolic acid (MPA)	4 (3.0)
Azathioprine (AZA)	15 (11.1)
Cyclosporine (CyA)	10 (7.4)
Corticosteroids only	34 (25.2)
Adalimumab monotherapy	53 (39.2)

n = 65 patients / 135 measurements at different timepoints. *Etiological categories were not mutually exclusive. VKH, Vogt–Koyanagi–Harada disease; IMT, immunomodulatory therapy (conventional); MTX, methotrexate; MPA, mycophenolic acid; AZA, azathioprine; CyA, cyclosporine; ADA, adalimumab; TDM, therapeutic drug monitoring.

Among patients with prior biologic exposure (n=20), 6 had received a prior anti-TNF agent (infliximab n=4, certolizumab n=1, etanercept n=1), while 14 had been treated with non-anti-TNF biologics (tocilizumab n=8, rituximab n=4, secukinumab n=2). Anti-drug antibodies to the prior biologic agent were not routinely measured before switching to adalimumab in most cases, which represents a limitation for interpreting the risk of cross-immunogenicity (28–30).

### Serum ADA levels and clinical response

Patients underwent between one and five TDM assessments during follow-up. The mean serum ADA level was 7.47 µg/mL (range, 0–29.1).

**Figure 1:**
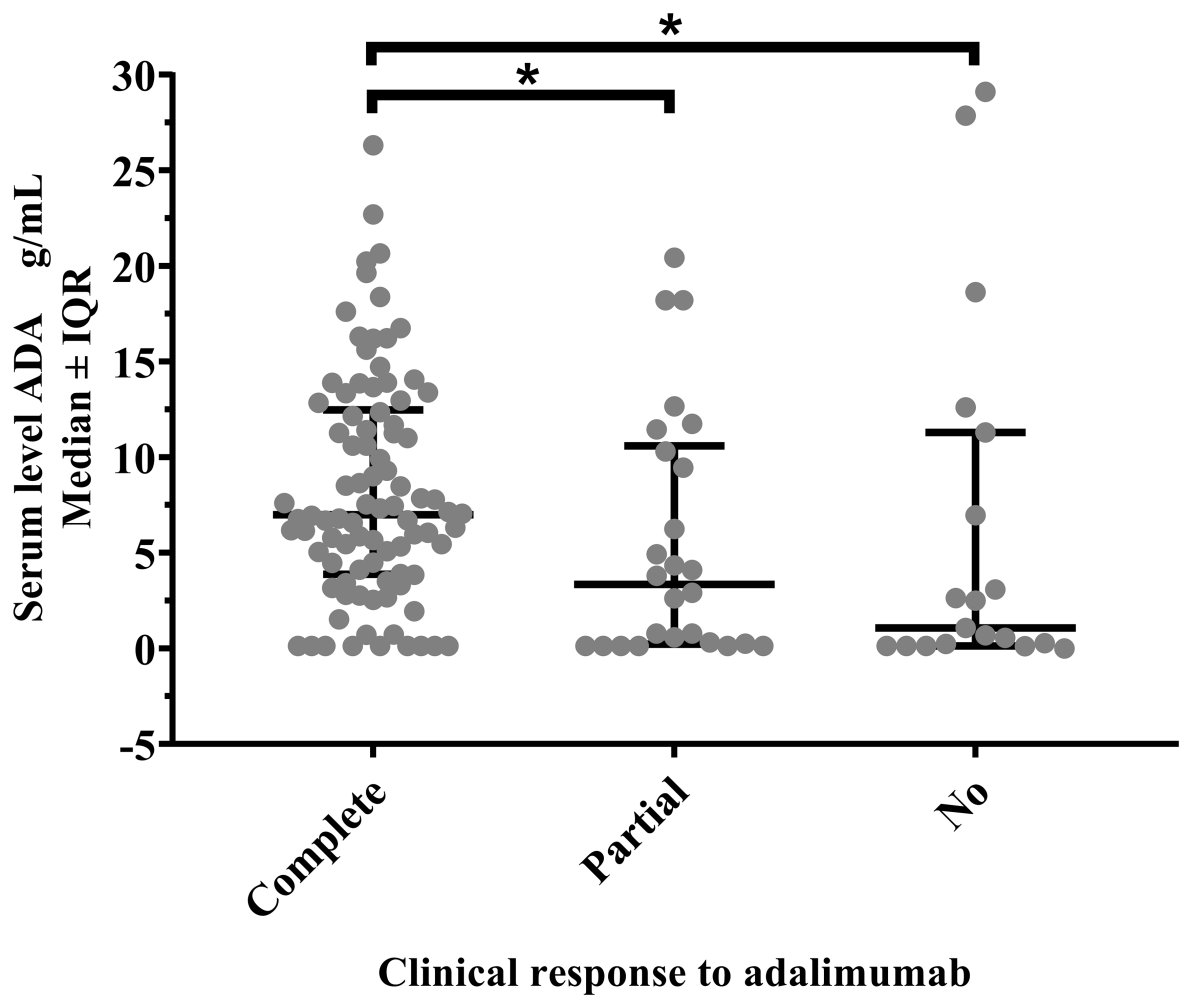
Serum trough adalimumab levels according to clinical response in non-infectious uveitis. Serum trough levels of adalimumab (ADA) in patients with non-infectious uveitis stratified according to clinical response (complete response, partial response, and no response). Each dot represents an individual measurement (n = 135). Horizontal lines indicate median values with interquartile range (IQR). Patients with complete response had higher ADA levels compared with partial responders and non-responders at the time of measurement. Statistical comparisons were performed using the Kruskal–Wallis test followed by *post hoc* pairwise comparisons. *p < 0.05.

Significant differences in serum ADA levels were observed across clinical response categories. Patients with complete response had higher mean ADA levels (8.28 µg/mL) compared with partial responders (5.57 µg/mL; p=0.016) and non-responders (6.21 µg/mL; p=0.012) (**Figure 1**).

These findings indicate an association between systemic ADA exposure and control of intraocular inflammation.

### Anti-adalimumab antibodies and their impact on drug exposure

AAA were detected in 13 patients (20%), corresponding to 18 of 135 measurements (13.3%). The mean AAA concentration was 571 AU/mL (range, 2.8–2000).

**Figure 2:**
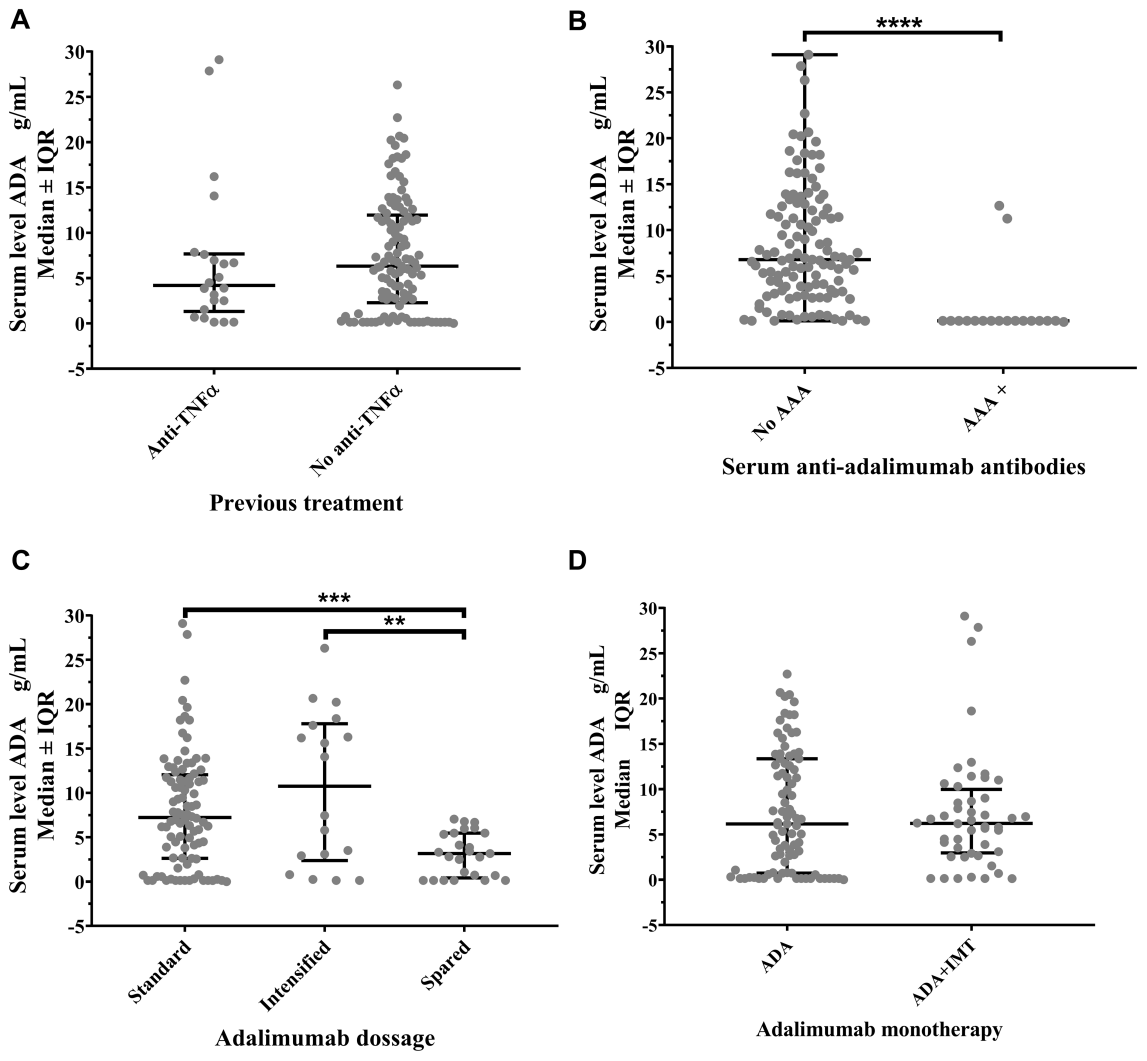
Determinants of serum adalimumab levels in patients with non-infectious uveitis. Serum trough levels of adalimumab (ADA) according to clinical and treatment-related variables in patients with non-infectious uveitis. **(A)** ADA levels according to previous exposure to anti-TNF therapy (anti-TNFα vs no prior anti-TNFα). **(B)** ADA levels according to the presence of anti-adalimumab antibodies (AAA− vs AAA+). **(C)** ADA levels according to ADA dosing regimen (standard, intensified, and spaced). **(D)** ADA levels according to treatment strategy (adalimumab monotherapy vs adalimumab combined with immunomodulatory therapy [IMT]). Each dot represents an individual measurement (n = 135). Horizontal lines indicate median values with interquartile range (IQR). Statistical analyses were performed using the Mann–Whitney U test or Kruskal–Wallis test, as appropriate, followed by *post hoc* pairwise comparisons when applicable. **p < 0.01, ***p < 0.001, ****p < 0.0001.

Patients with AAA had markedly lower serum ADA levels (mean, 1.44 µg/mL) compared with AAA-negative patients (8.39 µg/mL; p<0.0001) (**Figure 2**), indicating a strong association between immunogenicity and reduced drug exposure.

The median time from ADA initiation to AAA detection was 44.5 months. Most AAA-positive patients were not receiving concomitant immunomodulatory therapy at the time of detection (8/13 patients, 61.5%).

Among the 13 AAA-positive patients, 5 (38.5%) were classified as complete responders, 4 (30.8%) as partial responders, and 4 (30.8%) as non-responders. In comparison, among the 52 AAA-negative patients, 37 (71.2%) achieved complete response, 9 (17.3%) had partial response, and 6 (11.5%) were non-responders. The complete response rate was significantly lower in AAA-positive patients compared with AAA-negative patients (38.5% vs. 71.2%; p=0.02), confirming that immunogenicity is associated not only with reduced drug exposure but also with lower rates of clinical response.

Low-titer AAA were detected in two patients with preserved ADA levels (>10 µg/mL), suggesting that not all antibodies were neutralizing. In one patient, AAA were transient and disappeared in subsequent measurements without affecting serum ADA levels, indicating a possible dynamic immunogenic response over time.

Of the 13 patients with detectable AAA, 8 had follow-up TDM assessments available. Among these, AAA became undetectable in 2 patients (25%) on subsequent measurements (transient AAA), while AAA persisted or increased in titer in 6 patients (75%) (persistent AAA). In patients with transient AAA, serum ADA levels remained detectable (>5 µg/mL) even during the period of AAA positivity, suggesting that these antibodies had low neutralizing capacity. In contrast, all patients with persistent AAA developed undetectable ADA levels, and experienced loss of clinical response.

In 5 patients with persistent AAA, methotrexate was introduced (12.5–20 mg/week). AAA became undetectable in 3 of these cases; however, serum ADA levels remained undetectable in all but one patient. In this single case, the combination of methotrexate initiation and ADA dose intensification was associated with recovery of detectable ADA levels and clinical response. Despite these interventions, adalimumab was ultimately discontinued in most patients with persistent AAA and undetectable drug levels, highlighting the impact of immunogenicity on treatment sustainability (19, 31).

### Determinants of serum ADA levels

**Figure 3:**
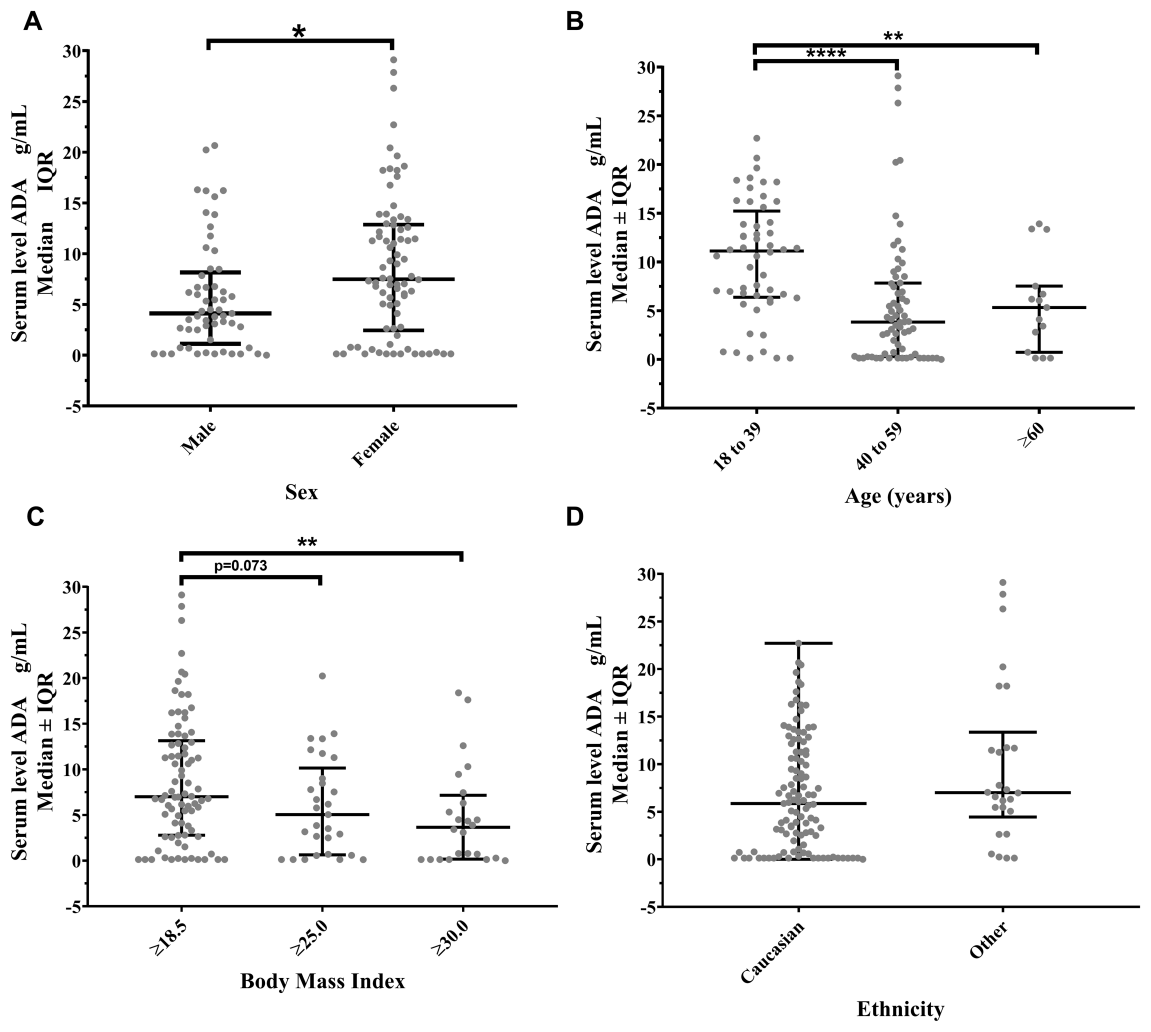
Association between patient characteristics and serum adalimumab levels in non-infectious uveitis. Serum trough levels of adalimumab (ADA) according to patient-related factors in non-infectious uveitis. **(A)** ADA levels according to biologic sex. **(B)** ADA levels according to age group. **(C)** ADA levels according to body mass index (BMI) categories (normal weight, overweight, and obesity). **(D)** ADA levels according to ethnicity. Each dot represents an individual measurement (n = 135). Horizontal lines indicate median values with interquartile range (IQR). Statistical analyses were performed using the Mann–Whitney U test or Kruskal–Wallis test, as appropriate, followed by *post hoc* pairwise comparisons when applicable. BMI categories defined as: normal (<25 kg/m²), overweight (25–29.9), obesity (≥30). *p < 0.05, **p < 0.01, ****p < 0.0001.

In bivariate analysis, several factors were associated with serum ADA levels. ADA levels were lower in male patients compared with females (5.75 vs. 8.72 µg/mL; p=0.011). Mean body weight was significantly higher in male patients compared with females (82.4 ± 14.2 kg vs. 66.8 ± 12.7 kg; p<0.001), and mean BMI was also higher in males (27.8 ± 4.1 kg/m² vs. 24.9 ± 4.5 kg/m²; p=0.003), suggesting that the observed sex-related difference in ADA levels may be confounded by differences in body size. Younger patients (18–39 years) had higher ADA levels compared with older age groups (p<0.01). Body mass index (BMI) was also associated with ADA levels, with lower concentrations observed in overweight and obese patients (**Figure 3**).

From a disease-related perspective, patients with panuveitis had lower ADA levels compared with other anatomical subtypes. No significant differences were observed between isolated ocular disease and uveitis associated with systemic conditions.

Variability in ADA levels was observed across etiological subgroups, with higher concentrations in internal punctate choroidopathy and serpiginous choroiditis, and lower levels in Behçet’s disease, birdshot chorioretinopathy, and Vogt–Koyanagi–Harada disease.

Previous treatment exposure was also associated with ADA pharmacokinetics. Patients naïve to systemic therapy before ADA initiation had higher serum ADA levels compared with those previously treated with corticosteroids, IMT, or other biologics (p<0.01 for all comparisons). No significant differences were observed between patients receiving ADA monotherapy and those receiving combination therapy at the time of sampling.

Dose-dependent effects were evident. Lower ADA levels were observed in patients undergoing dose spacing (mean, 3.12 µg/mL), whereas higher levels were found in those receiving intensified dosing (mean, 10.5 µg/mL).

**Figure 4:**
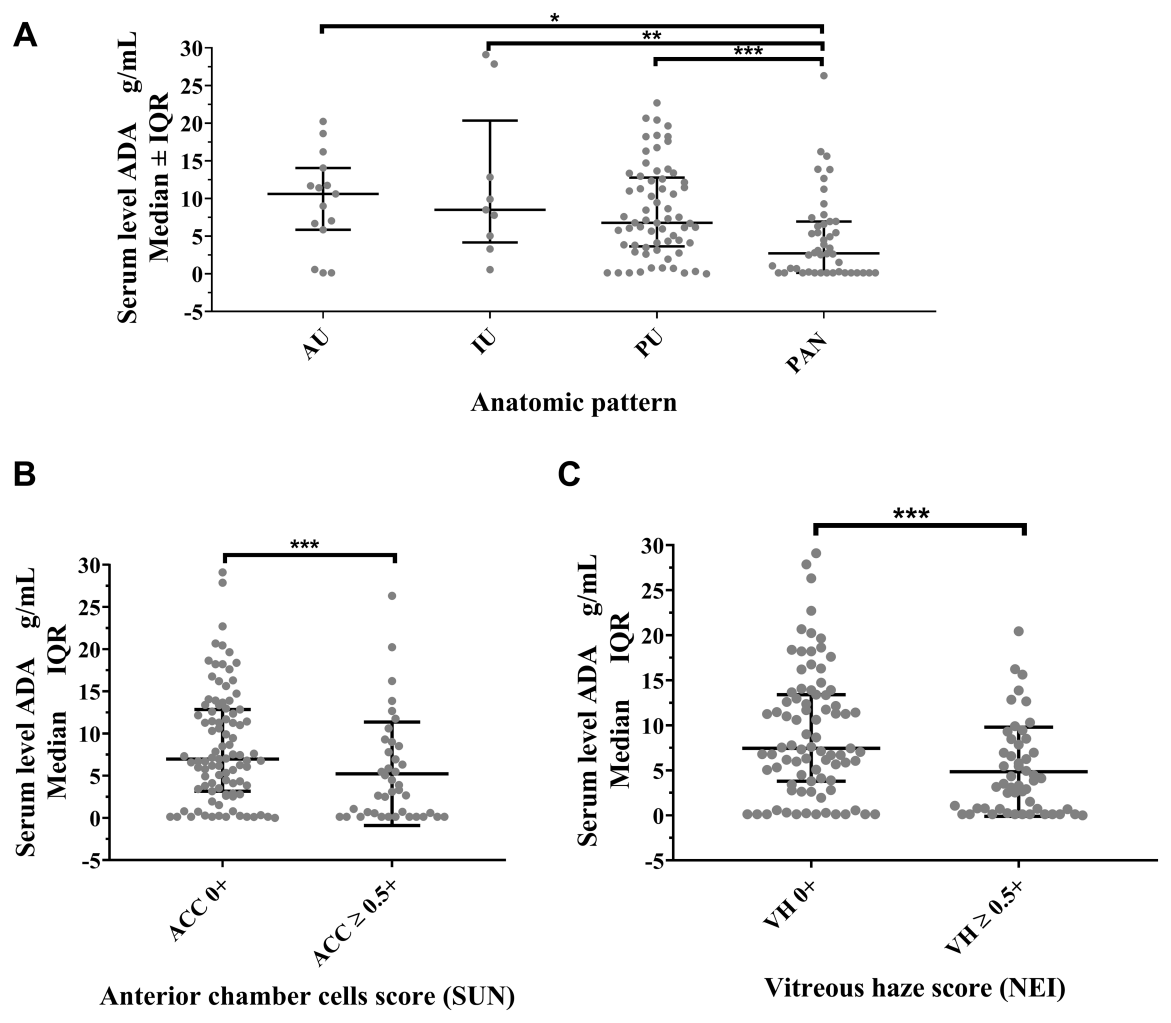
Association between inflammatory parameters and serum adalimumab levels in non-infectious uveitis. Serum trough levels of adalimumab (ADA) according to inflammatory disease characteristics in patients with non-infectious uveitis. **(A)** ADA levels according to anatomical classification of uveitis (anterior uveitis [AU], intermediate uveitis [IU], posterior uveitis [PU], and panuveitis [PAN]). **(B)** ADA levels according to anterior chamber cell grade based on the Standardization of Uveitis Nomenclature (SUN) criteria (0 vs ≥0.5+). **(C)** ADA levels according to vitreous haze grade based on the National Eye Institute (NEI) scale (0 vs ≥0.5+). Each dot represents an individual measurement (n = 135). Horizontal lines indicate median values with interquartile range (IQR). Statistical analyses were performed using the Mann–Whitney U test or Kruskal–Wallis test, as appropriate, followed by *post hoc* pairwise comparisons when applicable. *p < 0.05, **p < 0.01, ***p < 0.001.

Higher inflammatory burden, reflected by vitreous haze ≥0.5+, and longer disease duration were also associated with lower ADA levels (**Figure 4**).

In the LMM analysis, age, sex, and disease evolution time were included as covariates, because they were associated with serum adalimumab levels in univariable analyses. Patient identity was included as a random effect to account for repeated measurements within the same patient. Concomitant immunomodulatory therapy was evaluated in univariable analysis but was not significantly associated with serum adalimumab levels and was therefore not retained in the final multivariable model.

**Figure 5:**
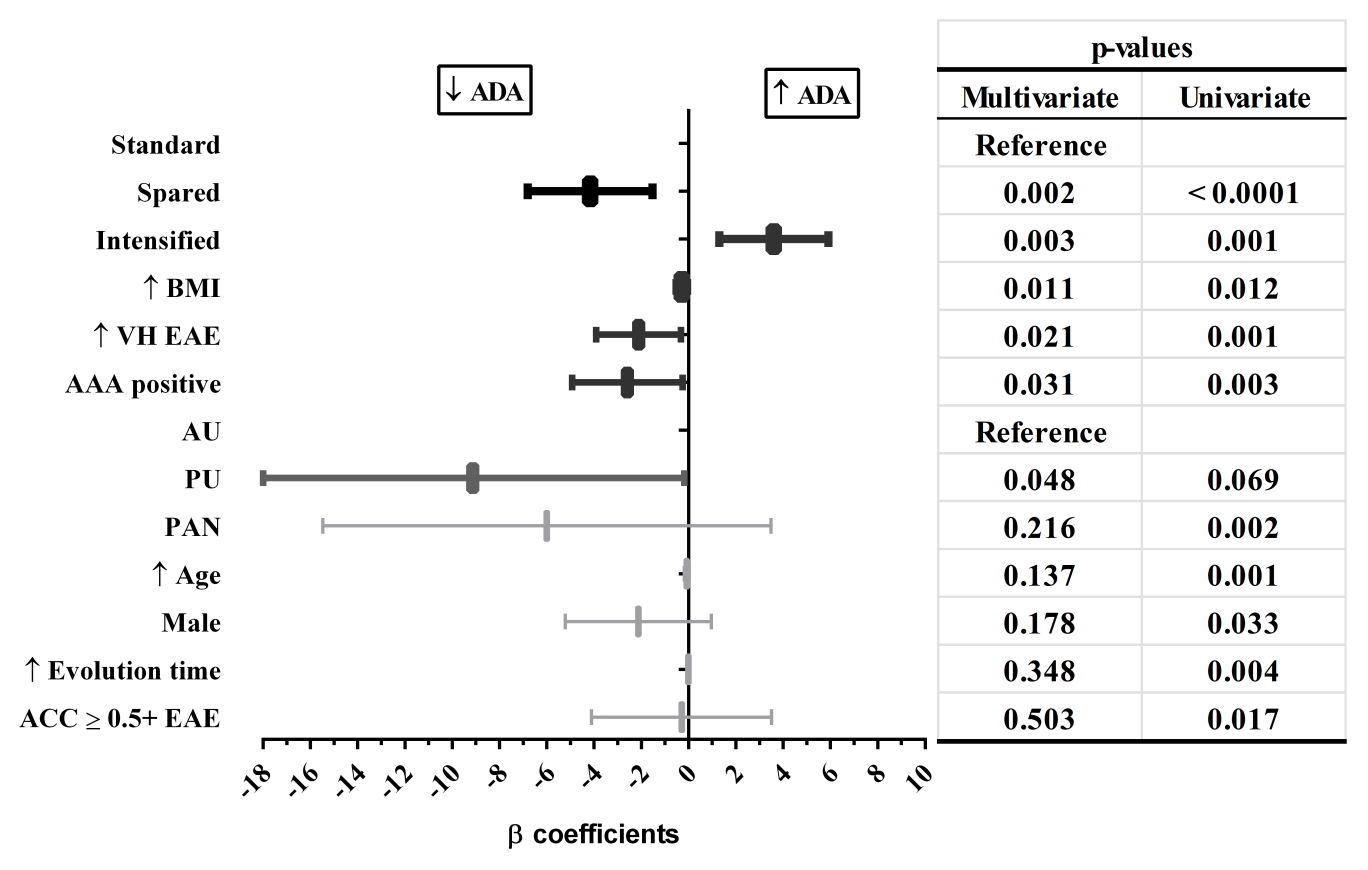
Linear mixed-effects regression analysis of factors associated with serum adalimumab levels. Estimates are presented as regression coefficients, or β coefficients, with 95% confidence intervals. Negative β coefficients indicate an association with lower serum adalimumab levels, whereas positive β coefficients indicate an association with higher serum adalimumab levels. Patient identity was included as a random effect to account for repeated measurements. ADA, adalimumab; BMI, body mass index; VH, vitreous haze; AAA, anti-adalimumab antibodies; AU, anterior uveitis; PU, posterior uveitis; PAN, panuveitis; ACC, anterior chamber cell score; EAE, either affected eye.

Finally, the LMM model showed BMI, vitreous inflammation, anatomical pattern of uveitis, presence of AAA, and ADA dosing regimen to be independently associated with the serum ADA levels (**Figure 5**).

### Association between LMM-identified factors and clinical response rates

To evaluate whether the factors independently associated with serum ADA levels in the LMM analysis also translated into differences in clinical response rates, we analyzed the proportion of complete responders across the relevant subgroups.

Complete response rates by anatomical classification were as follows: anterior uveitis 87.5% (7/8), intermediate uveitis 80.0% (4/5), posterior uveitis 68.0% (17/25), and panuveitis 55.6% (15/27) (p=0.14). Although panuveitis showed the lowest complete response rate, consistent with its association with lower ADA levels in the LMM model, the difference did not reach statistical significance, likely due to limited sample size in each subgroup.

Regarding BMI, complete response rates were 75.0% in patients with BMI <25 kg/m², 63.2% in those with BMI 25–30 kg/m², and 50.0% in those with BMI ≥30 kg/m² (p=0.09). AAA-positive patients had a lower complete response rate (38.5%, 5/13) compared with AAA-negative patients (71.2%, 37/52; p=0.02). Among dosing subgroups, patients on standard dosing had a complete response rate of 68.9%, compared with 83.3% for intensified dosing and 41.7% for dose-spaced regimens (p=0.04).

These findings suggest that factors associated with lower ADA levels in the LMM model show a trend toward lower clinical response rates, with statistically significant associations observed for AAA status and dosing strategy.

### Impact of therapeutic drug monitoring on clinical management

Therapeutic drug monitoring led to treatment modifications in a substantial proportion of patients. ADA was discontinued in 15 patients (23.1%), including one case of primary non-response and five cases of secondary loss of response. The reasons for ADA discontinuation in the remaining 9 patients were as follows: persistent AAA with undetectable drug levels despite clinical remission (n=3), adverse events (n=2), patient preference or non-compliance (n=2), and sustained remission allowing planned treatment withdrawal (n=2). In three patients with undetectable serum ADA levels due to AAA, treatment was discontinued despite clinical remission, and no relapses were observed during follow-up.

Dose adjustments were implemented in 17 patients (26.1%), including dose spacing (n=6) and dose intensification (n=11). In six patients, dose intensification during active disease was followed by clinical improvement.

Among the 11 patients who underwent dose intensification, the primary indications were: persistent or recurrent intraocular inflammation with subtherapeutic ADA levels (n=8), active disease with borderline ADA levels (n=2), and active disease with detectable AAA (n=1). The median time from ADA initiation to dose intensification was 18 months (range, 4–72). Of the 5 patients who did not achieve clinical improvement after dose intensification, 3 had persistent AAA with undetectable ADA levels despite intensification and were subsequently switched to an alternative biologic agent; 1 patient had adequate ADA levels but persistent inflammation suggestive of pharmacodynamic (mechanistic) failure and was switched to tocilizumab; and 1 patient had a partial but insufficient response and required the addition of a conventional immunomodulator.

Among the 6 patients who underwent dose spacing, the indication was sustained clinical remission with supratherapeutic or adequate ADA levels in all cases. The median time from ADA initiation to dose spacing was 24 months (range, 12–60). Two of these patients subsequently experienced disease relapse associated with a decline in ADA trough levels, requiring return to standard dosing intervals.

Together, these findings suggest that TDM contributed to individualized therapeutic decisions, including treatment escalation, de-escalation, discontinuation, and the interpretation of immunogenicity-related treatment failure.

## Discussion

Effective corticosteroid-sparing strategies are essential for achieving sustained disease control in non-infectious uveitis (NIU) while minimizing ocular damage and long-term corticosteroid-related toxicity. In this context, adalimumab (ADA), administered at a standard dose of 40 mg every two weeks, has demonstrated robust efficacy in controlling intraocular inflammation and reducing relapse rates. However, primary non-response and secondary loss of response remain clinically relevant challenges, underscoring the need to better understand the determinants of treatment variability, including pharmacokinetics and immunogenicity (32–34).

Therapeutic drug monitoring (TDM) has emerged as a valuable strategy to optimize biologic therapies across immune-mediated inflammatory diseases. In NIU, however, evidence remains limited and heterogeneous, and no universally accepted therapeutic range for ADA has yet been established. Our study provides real-world data from one of the largest single-center adult NIU cohorts undergoing proactive TDM, and the findings are consistent with recent ophthalmology literature suggesting that serum ADA levels and anti-adalimumab antibodies (AAA) are clinically relevant biomarkers in this setting (19–21, 35–40).

The optimal frequency of TDM in NIU has not been definitively established. While our proactive approach involved assessments every 3–6 months, the frequency should likely be individualized based on disease activity, treatment response, and immunogenicity risk. In patients with sustained disease control and therapeutic drug levels, less frequent monitoring (e.g., every 6–12 months) may be appropriate, whereas more frequent assessments may be justified in cases of active inflammation or suspected immunogenicity.

Consistent with observations in other immune-mediated diseases and prior uveitis studies, we observed an association between higher serum ADA levels and improved control of intraocular inflammation. NIUs with complete response had higher ADA levels than partial and non-responders, supporting a pharmacokinetic–pharmacodynamic association in NIU. This interpretation is further supported by studies showing that TDM can identify patients with subtherapeutic drug exposure, immunogenicity-related treatment failure, and candidates for dose optimization or switching (19–21, 35, 37, 39, 40).

From a mechanistic perspective, insufficient ADA levels may lead to incomplete TNF-α neutralization, allowing persistence of inflammatory pathways involved in leukocyte recruitment, vascular leakage, and tissue damage. Conversely, persistent inflammation despite adequate ADA levels may reflect pharmacodynamic failure or TNF-independent mechanisms, supporting a switch to an alternative therapeutic target rather than further ADA escalation. This distinction between pharmacokinetic and mechanistic failure represents one of the main practical advantages of TDM in clinical decision-making (37, 39, 40).

In our cohort, dose intensification in patients with active disease and low ADA levels was followed by clinical improvement in several cases, supporting the clinical utility of TDM-guided escalation. These findings are consistent with recent data by Pichi et al., demonstrating that treatment adjustment based on ADA levels and AAA status—particularly dose escalation combined with low-dose conventional immunomodulators—may improve outcomes in immunized non-responders (37). Similarly, other recent studies have supported the feasibility and clinical value of TDM-based approaches in routine management of NIU (39, 40).

Conversely, proactive TDM may help identify patients with sustained disease quiescence and low or undetectable ADA levels in whom continued treatment may not be necessary. In our series, some patients remained relapse-free after ADA discontinuation despite undetectable drug levels, suggesting that sustained remission may persist independently of ongoing TNF blockade in selected cases. This observation supports a potential role for TDM not only in treatment escalation but also in rational de-escalation and discontinuation strategies (37, 40).

AAA were detected in 13.3% of measurements and in 20% of patients, which is broadly consistent with previous uveitis studies. A recent systematic review and meta-analysis by Pachón-Suárez et al. highlighted that AAA are a clinically relevant and likely underrecognized phenomenon in NIU, with prevalence varying according to study design, assay methodology, and follow-up duration (36). Similarly, Bellur et al. demonstrated that anti-drug antibodies to TNF inhibitors are associated with reduced serum drug levels and treatment failure in NIU (24), while more recent studies have confirmed that higher AAA levels are associated with lower ADA concentrations and earlier loss of response (37–39).

These findings are biologically plausible. AAA may neutralize ADA activity or form immune complexes that accelerate drug clearance, leading to reduced circulating through levels and impaired TNF blockade. In our study, AAA positivity was strongly associated with lower serum ADA levels, consistent with previous reports and supporting the clinical relevance of immunogenicity assessment (19, 20, 24, 37–39).

The timing of AAA development remains variable. Earlier studies in NIU, particularly in pediatric JIA-associated uveitis, reported relatively early detection, whereas in our cohort the time to detection appeared longer. This difference likely reflects the retrospective design and timing of antibody assessment rather than true biological differences. Recent studies also suggest that transient AAA may occur and may not carry the same clinical significance as persistent antibodies, particularly when titers are low and serum ADA levels are maintained (36–38).

The role of concomitant immunomodulatory therapy in reducing immunogenicity remains uncertain. Some studies have suggested that methotrexate and other conventional agents may reduce the risk of AAA formation, although findings are inconsistent. In our cohort, the addition of methotrexate was associated with disappearance of AAA in several cases, although recovery of detectable ADA levels was limited. This observation aligns with recent data suggesting that concomitant therapy may reduce immunogenicity in selected patients but may not fully reverse established pharmacokinetic failure (18, 20, 23, 24, 37). The multifactorial nature of immunogenicity has been highlighted in recent studies. Bromeo et al. identified treatment interruption and dose-spacing as significant risk factors for AAA development in NIU, while other studies have suggested that low early drug exposure, irregular dosing, and absence of concomitant immunomodulatory therapy increase the risk of AAA formation (4, 8, 41).

It is important to acknowledge the possibility of reverse causation. Active ocular inflammation, particularly panuveitis and vitritis, may increase adalimumab clearance through antigen-driven drug consumption, protein loss, or inflammation-related immunogenicity. Therefore, the association between higher inflammatory burden and lower ADA levels may reflect inflammation-driven drug clearance rather than a direct causal effect of low ADA levels on disease activity. Recent studies similarly reported lower ADA levels in active disease and an association between disease activity and AAA formation (1–4). Longitudinal studies are needed to determine directionality, which could not be assessed in our retrospective cohort due to variability in TDM timing.

Beyond immunogenicity, several patient- and disease-related factors were associated with ADA exposure. Higher BMI was independently associated with lower serum ADA levels, a finding supported by recent NIU data showing that increased BMI is associated with lower drug exposure and higher disease activity (42). Increased inflammatory burden, reflected by vitreous inflammation and panuveitis, was also associated with lower ADA levels, suggesting increased drug clearance, or immunological sink phenomenon, in highly active inflammatory states (38–40, 42).

These findings are consistent with pharmacokinetic data from rheumatoid arthritis showing that adalimumab clearance is significantly higher in men, an effect largely attributable to differences in body weight rather than biological sex per se (43, 44). In our cohort, male patients had significantly higher body weight and BMI than female patients, and sex was not retained as an independent predictor in the multivariable model, further supporting the interpretation that body size, rather than sex itself, may drive the observed difference in ADA exposure.

We did not observe meaningful differences in ADA levels between isolated ocular disease and NIU associated with systemic immune-mediated disease. Existing evidence remains inconsistent, and our findings suggest that inflammatory burden and treatment-related factors may be more relevant determinants of drug exposure than the presence or not of an associated autoimmune systemic disease alone (36, 38, 41).

Cross-immunogenicity between sequential anti-TNF agents is well documented in inflammatory bowel disease and rheumatologic conditions, with patients who develop antibodies to one anti-TNF agent being at increased risk of developing antibodies to a subsequent anti-TNF. In our cohort, 6 patients had prior anti-TNF exposure, and they showed a significant lower ADA levels than anti-TNF-naïve in bivariate analysis; however, the lack of anti-drug antibody testing to the prior agent limits our ability to assess the contribution of cross-immunogenicity to AAA development in these patients.

As expected, dosing strategy had a direct impact on serum ADA levels. Dose spacing was associated with lower trough concentrations, whereas intensified regimens resulted in higher levels. These findings have practical implications, suggesting that TDM should be considered before treatment de-escalation to avoid subtherapeutic exposure. Recent studies support the use of individualized dosing strategies based on TDM to improve long-term outcomes (37, 39, 40, 42).

The retrospective design and inclusion of patients who underwent at least one TDM assessment may introduce selection bias. Although TDM was performed proactively at 3–6 month intervals or at the discretion of the treating physician, the indication for and timing of each assessment were not fully standardized, which may have influenced the observed results. Variability in follow-up intervals and individual clinical practice patterns may also have affected the interpretation of our findings. Additionally, the retrospective design and variable timing of TDM assessments precluded time-to-event analyses (e.g., time to AAA development, time to loss of response) that would have provided valuable insights into the temporal dynamics of immunogenicity and treatment failure. Future prospective studies with standardized TDM protocols should incorporate such analyses to better characterize the natural history of immunogenicity and loss of response in NIU.

Moreover, the predominantly Caucasian composition of our cohort (80%) limits the generalizability of our findings to more ethnically diverse populations. While genetic factors, particularly HLA alleles, have been associated with immunogenicity to adalimumab and may vary in frequency across populations, evidence regarding clinically significant racial or ethnic differences in adalimumab outcomes in NIU is lacking. Multicenter studies with greater ethnic diversity would be valuable to evaluate potential differences in pharmacokinetics and immunogenicity across populations (30, 31, 45, 46).

Although repeated measurements were accounted for in the multivariable analysis by including patient identity as a random effect, repeated measurements were not modeled as a random effect in all bivariate analyses. Therefore, these exploratory comparisons should be interpreted with caution. In addition, differences in assay methodologies remain a major source of variability across studies and limit the definition of a standardized therapeutic range (47–48). These limitations have also been highlighted in recent TDM-focused studies in NIU (36, 38–40).

In conclusion, our findings support an association between serum ADA levels and inflammatory control in NIU and highlight immunogenicity as a key determinant of treatment variability. Proactive TDM may help differentiate pharmacokinetic underexposure from potential mechanistic failure, guide dose optimization or treatment switching, and support treatment de-escalation in selected patients. Taken together with recent literature, these results support a more personalized, biomarker-driven approach to ADA therapy in NIU (49–54).

## Data Availability

The original contributions presented in the study are included in the article/supplementary material. Further inquiries can be directed to the corresponding author.
